# Multifunctional Aspects of Mechanical and Electromechanical Properties of Composites Based on Silicone Rubber for Piezoelectric Energy Harvesting Systems

**DOI:** 10.3390/polym16142058

**Published:** 2024-07-18

**Authors:** Vineet Kumar, Md. Najib Alam, Manesh A. Yewale, Sang-Shin Park

**Affiliations:** School of Mechanical Engineering, Yeungnam University, 280 Daehak-Ro, Gyeongsan 38541, Gyeongbuk, Republic of Korea; vineetfri@gmail.com (V.K.); mdnajib.alam3@gmail.com (M.N.A.); maneshphd@gmail.com (M.A.Y.)

**Keywords:** titanium carbide, molybdenum disulfide, silicone rubber, reinforcing efficiency, electromechanical properties

## Abstract

Energy harvesting systems fabricated from rubber composite materials are promising due to their ability to produce green energy with no environmental pollution. Thus, the present work investigated energy harvesting through piezoelectricity using rubber composites. These composites were fabricated by mixing titanium carbide (TiC) and molybdenum disulfide (MoS_2_) as reinforcing and electrically conductive fillers into a silicone rubber matrix. Excellent mechanical and electromechanical properties were produced by these composites. For example, the compressive modulus was 1.55 ± 0.08 MPa (control) and increased to 1.95 ± 0.07 MPa (6 phr or per hundred parts of rubber of TiC) and 2.02 ± 0.09 MPa (6 phr of MoS_2_). Similarly, the stretchability was 133 ± 7% (control) and increased to 153 ± 9% (6 phr of TiC) and 165 ± 12% (6 phr of MoS_2_). The reinforcing efficiency (R.E.) and reinforcing factor (R.F.) were also determined theoretically. These results agree well with those of the mechanical property tests and thus validate the experimental work. Finally, the electromechanical tests showed that at 30% strain, the output voltage was 3.5 mV (6 phr of TiC) and 6.7 mV (6 phr of MoS_2_). Overall, the results show that TiC and MoS_2_ added to silicone rubber lead to robust and versatile composite materials. These composite materials can be useful in achieving higher energy generation, high stretchability, and optimum stiffness and are in line with existing theoretical models.

## 1. Introduction

Piezoelectric energy harvesting is a novel technique that can convert kinetic energy into an electric charge using piezoelectric materials. Integrating this technology into composite materials leads to multifunctional possibilities, such as creating flexible, durable, stretchable, and lightweight materials [1]. Piezoelectric materials commonly involve ceramics like lead zirconate titanate (PZT) and polymer matrices like polyvinylidene fluoride (PVDF). For rubber composites, dielectric rubbers like silicone rubber are mixed with piezoelectric fillers like PZT [2,3]. This integration allows the composite to maintain the flexibility and durability of the rubber while gaining the ability to harvest energy. There are various applications for piezoelectric rubber composites. They include wearable electronics, structural health monitoring, energy harvesting mats, and automobile applications. Wearable electronics are used in power sensors and portable electronics through human motion [4]. Energy harvesting mats are embedded flooring materials that capture energy from foot traffic. This mechanical energy, obtained through a foot press, is converted from footsteps into electric power [5]. However, there are various limitations to using these rubber composites for energy harvesting. These include poor efficiency, a low coupling coefficient, low power density, scalability, and integration [6]. Thus, seamlessly integrating these composites into existing systems and ensuring their compatibility with other materials and components is crucial before commercialization. Overall, this piezoelectric energy harvesting system with rubber composites provides a promising area for research and development, with potential applications across various industries [7].

The mechanical properties of these piezoelectric rubber composites are crucial for their performance and multifunctional applications. These properties determine the composite material’s behavior under mechanical deformation. They also determine the ability of these composites to maintain their piezoelectric features under cyclic mechanical deformation [8]. Many mechanical parameters, such as the modulus, tensile strength, fracture toughness, and elongation at break, are critical for the overall performance of the composite. For example, for piezoelectric rubber composites, achieving a balance between flexibility (low elastic modulus) and sufficient mechanical strength is essential [9,10]. Moreover, the tensile strength and elongation at break are critical for applications that involve stretching or bending, ensuring that the composite can endure mechanical stress without failure. These mechanical properties are influenced by various factors. These factors include the filler loading, the type of rubber matrix, filler dispersion, filler–rubber compatibility, and filler–rubber interfacial interactions [11]. A balance among these factors is required to obtain robust energy harvesting performance. Overall, understanding and optimizing these mechanical properties of piezoelectric rubber composites is crucial for their effective application in various fields. These applications include strain sensors, actuators, and smart textiles [12].

The electromechanical properties of piezoelectric rubber composites are characterized by the interaction between mechanical deformation and an electrical response. This piezoelectric effect can be direct or inverse depending upon the required application [13]. The direct effect converts mechanical deformation into an electrical response, such as in energy harvesting. However, the inverse effect converts electrical energy into mechanical displacement, as in actuators. Moreover, mechanical properties such as stiffness, flexibility, stretchability, and fatigue resistance are very important in influencing the electromechanical properties [14]. Various key factors influence the electromechanical properties. These are the nature of the piezoelectric material, the type of rubber matrix, filler dispersion, filler–rubber interfacial adhesion, and filler–rubber compatibility [15]. For example, the filler–rubber interfacial bonding assists in enhancing the stress transfer from the rubber matrix to the filler particles when subjected to mechanical deformation. In addition, filler–rubber compatibility is very important as it strongly influences the filler dispersion and interfacial bonding in composites [16]. Moreover, the processing technique for the fabrication of composites also critically influences the electromechanical behavior. Overall, the electromechanical properties of piezoelectric rubber composites are critical to their functionality and application potential. These applications include sensors, energy harvesting, actuators, and health monitoring systems [17,18].

Various studies demonstrate the use of rubber composites for energy harvesting applications [19,20,21,22]. For example, Liu et al. [19] studied composites based on lead zirconate titanate, graphene, and polydimethylsiloxane as a rubber matrix. The results show that a high dielectric constant of 30 was achieved. These improved electrical properties helped in achieving an output voltage of ~40 mV using a universal testing machine and ~3 V through human motion. In another interesting study by Kumar et al. [20], the composites were prepared by mixing silicone rubber with barium titanate, carbon black, and multiwall carbon nanotubes. The composites fabricated exhibited high tensile strength of 0.2 MPa, hardness of 33, and electrical resistance of 260 Ω. The energy harvesting properties showed an output voltage of ~1.8 V at an electrical poling value of 10 kV/mm. In another study, Deng et al. [21] reinforced silicone rubber with carbon nanotubes and achieved great mechanical stretchability of ~900%. The authors achieved a gauge factor of 2.1 at 130% and finally a power density of 21.7 W cm^−1^. Lastly, Chung et al. [22] reported on composites that were prepared by mixing poly[styrene-b-isoprene-b-styrene] with carbon black and multiwall carbon nanotubes. The results showed that a robust stretching ability of >1000% was obtained. Moreover, a gauge factor of 69.8 at ~450% strain and durability of 10,000 cycles were reported. In addition, the best output voltage of 0.5 V, electrical resistance of 100 Ω, and durability of >25,000 cycles were reported [22].

With these aspects in mind, the present work involves the fabrication of composites. A comparative study on electromechanical properties like the response time and sensitivity for silicone-rubber-reinforced titanium carbide (TiC) and molybdenum disulfide (MoS_2_) is performed for the first time. Moreover, the reinforcing factor, reinforcing efficiency, and their multifunctionalities are reported for silicone rubber with TiC and MoS_2_ as they are also not well understood yet. Besides this, the study focuses on harvesting mechanical energy through the piezoelectric principle. The results show a robust improvement in the stretchability of the composites. For example, the stretchability is 125% (control) and increases to 153% (6 phr of TiC) and 165% (6 phr of MoS_2_). The electromechanical results show that the output voltage is 3.5 mV (6 phr of TiC) and 6.7 mV (6 phr of MoS_2_). Moreover, the theoretical models show that the experimental modulus is in fair agreement with the filled composites up to 6 phr and then deviates. Finally, the biomechanical results show that the output voltage is the highest for thumb pressing. For example, thumb pressing results in an output voltage of ~10 mV (6 phr of TiC) and 5.6 mV (6 phr of MoS_2_). Thus, this paper is novel as it describes various aspects that are not understood fully in the literature on TiC- and MoS_2_-based silicone rubber composites. Therefore, they are reported in the present work experimentally and validated theoretically.

## 2. Experimental Section

### 2.1. Materials and Methods

A transparent room-temperature-vulcanized silicone rubber with the commercial name “KE-441-KT” was used as a rubber matrix. A curing agent with the commercial name “CAT-RM” was used. Both the rubber and curing agents were purchased from the Shin-Etsu Chemical Corporation, Japan. A metal-like ceramic and 3-dimensional (3D) material named “titanium carbide (TiC)” was purchased from Sigma-Aldrich, Saint Louis, MI, USA. Two-dimensional (2D) molybdenum disulfide (MoS_2_) with a particle size of ~2 µm and chemical purity of >98% was bought from Sigma-Aldrich, USA. TiC and MoS_2_ were used as reinforcing fillers and to improve the electrical conductivity. Finally, an anti-adhesive spray was sprayed on the molds as a mold-releasing agent and was purchased from Nabakem, Pyeongtaek-si, Korea.

### 2.2. Preparation of Rubber Composites

The preparation of rubber composites is a very important step in influencing the mechanical and electromechanical properties of the composites. This fabrication step was optimized from a previous study and used without further modification [23]. The steps of preparation were as follows.

(1) As detailed in Table 1 below, different amounts of the filler and silicone rubber were taken and mixed for nearly 10 min through the solution mixing technique.

(2) Then, a known amount of curing agent was added and mixed for 1 min. The final composites were placed inside the molds and kept for 24 h for the vulcanization process. This vulcanization process was performed in a room-temperature vulcanization system at 25 °C.

(3) The final vulcanized composites were taken out of the molds and tested for their properties and application prospects.

### 2.3. Characterization Techniques

The mechanical property tests were performed under different types of strain, which were the compression and tensile modes. These tests were undertaken under a load cell of 1 kN using a universal testing machine (UTS, Lloyd instruments, Bognor Regis, UK). The static and cyclic compressive tests were performed on cylindrical samples with a diameter of 20 mm and thickness of 10 mm. The compressive tests were performed at a strain rate of 4 mm/min, pre-load of 0.5 N, and up to the maximum compressive strain of 35% for static and 30% for cyclic tests.

**Scheme 1:**
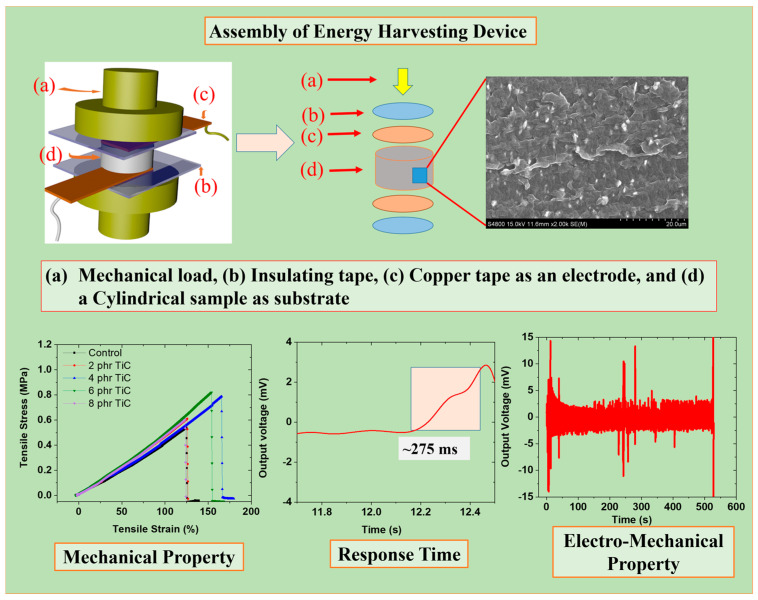
Assembly of energy harvesting devices and insights into their properties.

Other static tests were performed under uniaxial tensile strain at a pre-load of 0.1 N. The strain rate was 200 mm/min and dumbbell-shaped samples were used with a thickness of 2 mm, gauge length of 20 mm, and width of 4 mm. These mechanical tests were performed following the DIN 53 504 standard [24]. The electromechanical properties were also tested using the UTS machine on cylindrical samples under compressive cyclic deformation. These tests were performed at 10–30% cyclic strain using cylindrical samples. The mechanical and electromechanical long-term stability was tested at 30% compressive cyclic strain using the UTS machine. The output voltage generated through piezoelectricity was recorded using a multimeter (model 34401A, Agilent Technologies Inc., Santa Clara, CA, USA). The assembly of the energy harvesting set-up is provided in Scheme 1. Finally, the biomechanical properties were studied through real-time monitoring using a thumb press, index finger press, and middle finger press. For real-time monitoring, a cylindrical sample was used and the output voltage generated through piezoelectricity was recorded using the multimeter, as detailed above. Finally, the filler dispersion was studied using SEM (model S-4800, Hitachi, Tokyo, Japan). The fractured surface of the composite was sectioned into 0.2-mm-thick samples using a surgical blade. These sections were then mounted on an SEM stub and sputtered with platinum for 2 min before recording the SEM micrographs.

## 3. Results

### 3.1. Mechanical Properties under Compressive and Tensile Strain

The mechanical properties of composite materials are fundamentally important for various engineering applications. These mechanical properties help us to understand a composite material’s behavior under external forces or loads [25]. Moreover, the testing of these properties is very important depending on the nature of the strain under which they are tested. Most often, these strains are compressive or tensile strains, and different mechanical parameters, like the compressive modulus, tensile strength, or elongation at break, are tested [26]. The behavior of the composite materials under a mechanical load is often described through stress–strain curves. Moreover, understanding the mechanical properties is essential in optimizing the type of material, the fabrication process, and the prediction of the overall performance [27]. Thus, the mechanical properties provide the basis for the analysis and design of materials as per the application demands.

**Figure 1:**
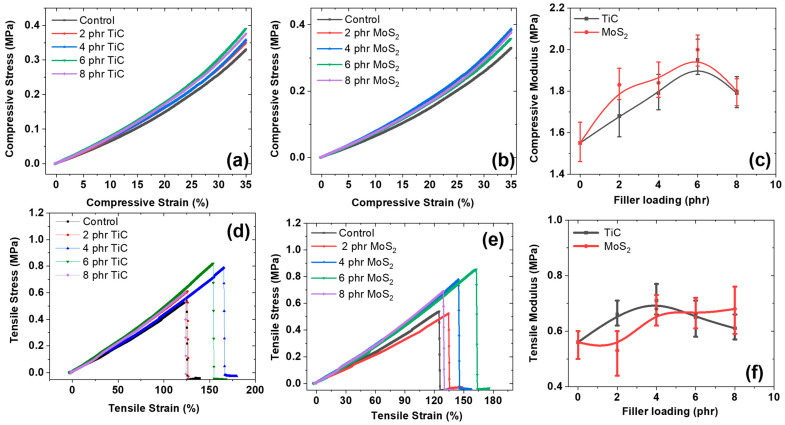
(**a**,**b**) Compressive stress–strain curves of TiC and MoS_2_ composites; (**c**) compressive moduli of TiC and MoS_2_ composites; (**d**,**e**) tensile stress–strain of TiC and MoS_2_ composites; (**f**) tensile moduli of TiC and MoS_2_ composites.

Figure 1a,b present the stress–strain curves for the prepared composites. The results show that the stress was higher for increasing strain until the compressive strength. Understanding this behavior involves examining how the material deforms under applied forces or compressive strain. The results show that the TiC and MoS_2_ act as reinforcing agents and their addition leads to an increase in the mechanical properties. Here, the rubber matrix is a continuous phase that holds these reinforcing fillers within the composite material. These reinforcing properties of the composites are mainly proposed to be due to filler–rubber interaction [28,29]. Such reinforcing effects of TiC and MoS_2_ contribute to improved mechanical properties and thus make them useful for high-load-bearing applications. The main mechanism of these reinforcing properties is to transfer the load from the matrix to the stronger, stiffer reinforcement [30]. These reinforcements are TiC and MoS_2_ as the fillers and SR as the rubber matrix. The efficiency of load transfer depends on the interface between the matrix and the reinforcement. The further reinforcement aspects are presented in Figure 1c through the compressive moduli of the composites. All values are reported in Table 2 below. The results show that the reinforcing effects of the MoS_2_- and TiC-filled composites are close and nearly the same under compressive strain. However, the control sample without fillers has the lowest compressive modulus. The good compressive moduli of the MoS_2_- and TiC-filled composites are due to their improved reinforcing effect in the rubber matrix. These reinforcing effects can help in arresting crack propagation within the matrix [31]. The TiC and MoS_2_ particles are proposed to deflect cracks, increasing the composite’s toughness to that of the control sample without fillers.

The mechanical properties can be further studied through stress–strain curves under tensile strain. Figure 1d,e show that the composites filled with MoS_2_ and TiC help in enhancing both the tensile strength and elongation at break as compared to the control sample without fillers. At nearly 6 phr, both fillers exhibit the best tensile strength and elongation at break and fall at 8 phr in the rubber composites. Thus, the inclusion of TiC and MoS_2_ significantly increases the mechanical properties. The degree of improvement in the mechanical properties depends on the type, orientation, and volume fraction of the fillers in the rubber matrix [32]. The higher reinforcing effect of MoS_2_ and TiC makes the composites stiff, rendering them less prone to deformation under a load. Moreover, these reinforcing agents further improve the ability of a composite to absorb energy and resist fracture, thereby making the composites tough. These tougher composites help to enhance the mechanical properties through mechanisms like crack bridging and deflection [33,34].

**Scheme 2:**
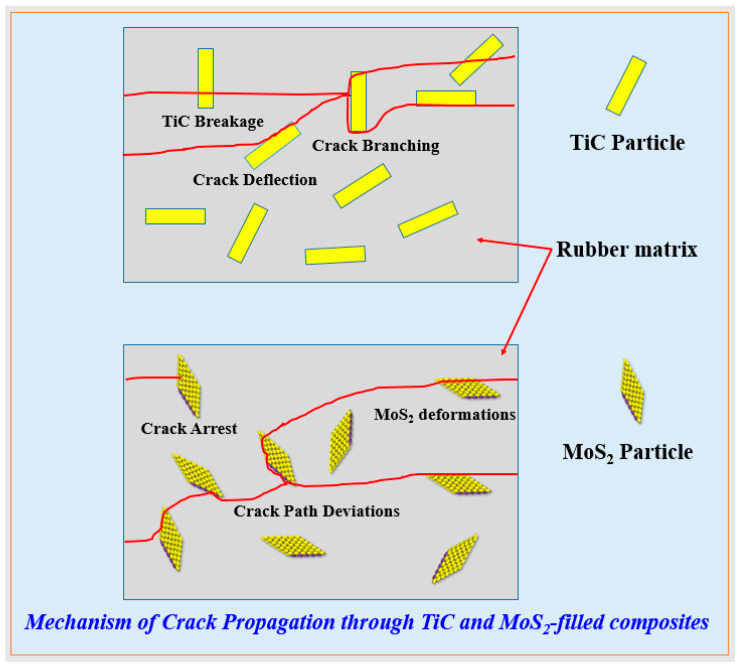
Crack propagation configurations for different composites.

The stiff and tougher composites are also examined for their tensile moduli, as presented in Figure 1f. The results agree with those of the compressive modulus, in which the filled composites exhibit higher tensile moduli than the control sample. All values are reported in Table 2 below. Fillers like TiC and MoS_2_ can help to redistribute the stress concentrations within the matrix, reducing the likelihood of failure under applied loads. Thus, TiC and MoS_2_ are expected to exhibit higher reinforcing effects than the control composite without fillers. However, the tensile modulus falls after 6 phr for TiC and is almost the same for the MoS_2_-filled composites. This is expected due to the partial aggregation of TiC, while there is almost a saturation effect with the MoS_2_-filled composites. Therefore, the reinforcing properties of composites are integral to their performance and versatility in rubber composites [35]. Here, the reinforcing effect of fillers like TiC and MoS_2_ leads to promising mechanical properties. Moreover, understanding and optimizing the interaction between the matrix and reinforcing fillers is important. Thus, engineers can design composites tailored to meet specific demands across various industries [36]. Moreover, under long cycling conditions, some cracks are developed, thereby leading to poorer properties. The mechanism of such crack propagation configurations for these composites is provided in Scheme 2.

### 3.2. Mechanical Properties under Compressive Cyclic Loading

**Figure 2:**
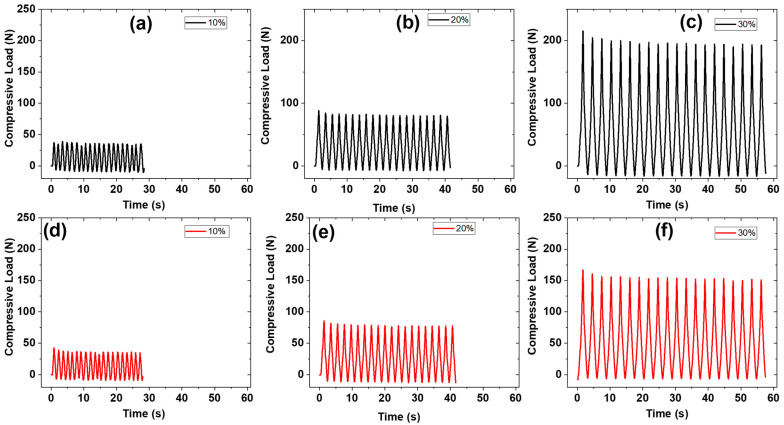
Mechanical properties under compressive cyclic strain at increasing strain from 10 to 30% for (**a**–**c**) TiC-based composites; (**d**–**f**) MoS_2_-based composites.

Compressive cyclic loading is very useful in studying fatigue properties and involves applying repeated compressive forces to a composite rubber material. This type of cyclic loading can lead to material fatigue, which is progressive and localized structural damage. The fatigue life is the number of cycles that a material can withstand before failure occurs under cyclic loading [37,38]. It is influenced by several factors, including the magnitude of the stress, the stress range, and the frequency of the cycles. This fatigue behavior generally occurs when a material is subjected to cyclic loading [39]. This type of compressive loading is common in many engineering applications, including structural components and automotive parts, including vibrational loads. Thus, in addition to the static tests described in Figure 1, cyclic loading tests are also important to understand the mechanical stability of the composites. Here, Figure 2a–c show the compressive load at the different strain magnitudes for 6 phr filler loading in the TiC-reinforced composites under compressive cyclic loading. The compressive load increases with increasing compressive strain for both fillers. For example, the compressive load is 40 ± 3 N (10%-TiC), 87 ± 5 N (20%-TiC) and 224 ± 16 N (30%-TiC). Similarly, the compressive load is 36 ± 3 N (10%-MoS_2_), 84 ± 6 N (20%-MoS_2_) and 170 ± 14 N (30%-MoS_2_). This compressive process involves the application of forces or strains that act to compress or shorten a material. This process is opposite to tensile loading, which pulls and stretches the material [40,41].

Moreover, cyclic loading refers to the repeated application and removal of loads over time and it can be symmetric or asymmetric. The results show that the compressive load is higher with increasing strain from 10% to 30%. The high load at higher strain is related to the packing fraction of filler particles in the rubber matrix [42]. For example, the packing is lower at 10%, higher at 20%, and highest for 30% strain. Moreover, a significant increase in compressive load by an order of magnitude was witnessed when the compressive strain increased from 20% to 30%. This can be postulated as the attainment of the optimum packing fraction of filler particles at 30% compressive strain. The results also show a higher compressive load at the first cycle and it then stabilizes in successive cycles. Therefore, the stabilized successive cycles suggest stable mechanical behavior and are beneficial for the durability and fatigue properties under a continuous compressive cyclic process [39,43].

Similarly, Figure 2d–f show the behavior of the compressive load with increasing compressive cyclic strain at 10–30% for the MoS_2_-reinforced composites. The results show that the compressive load was higher with increasing compressive strain. These results are proposed to be due to the influence of the packing fractions of filler particles in the rubber composites [42]. Moreover, as discussed above, the compressive load was significantly higher when it increased from 20 to 30% strain. These features are proposed to be due to the favorable packing, which results in a higher compressive load at 30% compressive strain. This behavior is especially important in applications where materials experience fluctuating loads, such as in bridges, buildings, and various mechanical components. Therefore, understanding the mechanical properties under compressive cyclic loading is essential [44,45]. These tests are useful in designing and maintaining components that experience repeated stress. It involves a complex interplay among the material properties, microstructural changes, and load conditions.

### 3.3. Electromechanical Properties under Compressive Cyclic Loading

As discussed already, compressive cyclic loading involves applying repeated compressive forces to a composite material or device. This type of loading can affect both the mechanical and electrical properties of electromechanical materials. The electromechanical properties in composite materials are related to both electrical and mechanical behaviors [46,47]. These properties are crucial in understanding how these rubber composite materials respond to repeated compressive loads. These electromechanical behaviors refer to the interaction between electrical and mechanical states in a composite material or device. The electroactive materials that exhibit this coupling include piezoelectric materials, electrostrictive materials, and magnetostrictive materials [48,49]. These properties are particularly significant in applications such as sensors, actuators, and various electronic components that experience cyclic mechanical stress.

**Figure 3:**
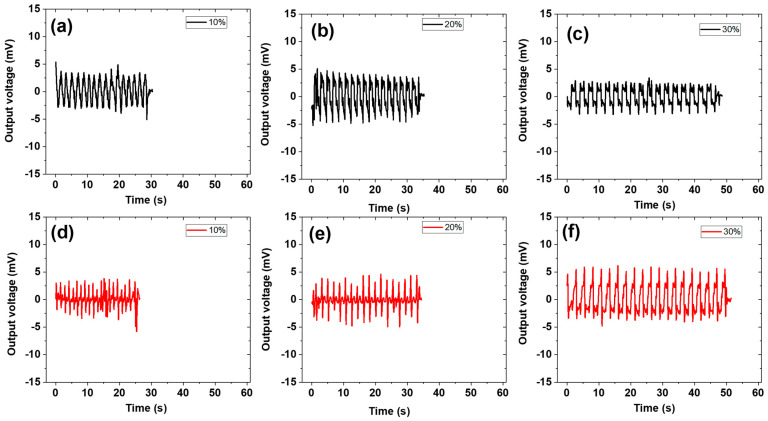
Electromechanical properties under compressive cyclic strain with increasing strain from 10 to 30% for (**a**–**c**) TiC-based composites; (**d**–**f**) MoS_2_-based composites.

With the above points in mind, Figure 3a–c present the behavior of the output voltage under increasing compressive cyclic strain from 10% to 30% for the 6 phr TiC. The results show that the output voltage was higher for higher compressive loads up to 20% strain and then decreases at 30% and above. This electromechanical behavior of TiC was slightly different from that of the compressive loads in Figure 2. This difference is proposed to be due to the electrical response, which causes the output voltage to be different from the mechanical behavior [50]. This electrical response is optimum at 20% compressive strain. The testing also involves applying repeated compressive loads to measure changes in the mechanical and electrical properties over time. Thus, both electrical and mechanical stability are important for electromechanical tests. Therefore, understanding the electromechanical properties under compressive cyclic loading is crucial in designing and maintaining reliable electromechanical devices [51,52].

Similarly, Figure 3d–f show the electromechanical behavior for the MoS_2_-filled composites at 6 phr. These tests were performed under compressive cyclic loading by increasing the strain from 10 to 30%. Therefore, a higher output voltage at 30% compressive strain was witnessed. These results are proposed to be due to the optimum packing fraction of the MoS_2_-filled composite at 30% strain, which results in an improved electrical and mechanical response. The results agree well with Figure 2, in which the mechanical behavior increases with increasing compressive strain. Overall, the electromechanical properties of materials under compressive cyclic loading are essential in achieving good performance that integrates electrical and mechanical properties [53]. The results also show that the selection of the appropriate filler and rubber materials and their optimum processing techniques are critical to obtaining robust electromechanical performance. The usefulness of these studies for various industrial applications extends to piezoelectric sensors, actuators, and energy harvesting devices [54]. The uses of these materials also include medical and sensing monitoring, where they can experience repeating mechanical stress. Finally, piezoelectrical energy harvesting in these composites involves the conversion of a mechanical load into a useful output voltage under compressive cyclic loading [55].

### 3.4. Response Time of Composites

The addition of fillers in the rubber matrix adds various functionalities to these composite materials. One of the critical functionalities of these rubber composites is their response time [56]. The response time refers to how quickly a material reacts to an applied force, deformation, or external stimulus, which is crucial in applications requiring timely and precise reactions. This response can be apparent in various ways depending on the type of application, such as deformation, vibration damping, or electrical signal generation [57]. The key factors influencing the response time include the material composition, method of fabrication, filler dispersion, and load parameters or type of deformation.

**Figure 4:**
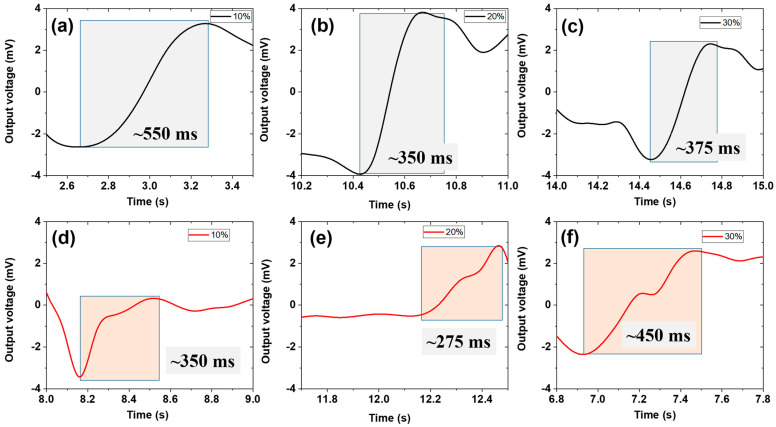
Response times of the composites at 6 phr filler loading, derived from electromechanical properties at different strains from 10 to 30%, for (**a**–**c**) TiC-based composites; (**d**–**f**) MoS_2_-based composites.

Among them, the type of mechanical response refers to the immediate, reversible deformation to external stimuli. Moreover, filled rubber composites generally exhibit viscoelastic behavior [58]. This behavior results from time-dependent deformation that includes both elastic and viscous components, leading to hysteresis and energy dissipation. However, the response rate studied in this work is related to electromechanical features, i.e., the influence of the output voltage against external stimuli [59]. Therefore, Figure 4a–c show the response times of composites filled with 6 phr of TiC under increasing strain of 10–30%. The results agree with the electromechanical results in Figure 3. For example, the response time was shortest at 20% compressive strain, as expected. This feature is proposed to be due to the optimum packing fraction of the filler particles at this strain [42]. These values further show that our results are consistent with each other. The mechanism behind these electromechanical responses refers to the generation of an electric charge in response to mechanical deformation [59]. Moreover, the process also involves the ability of the composite material to store or dissipate electrical energy in the form of an output voltage. Various factors affect the response time, and the type of filler is one of the most crucial factors [60]. The material type is well known to alter the response time by changing the composite’s mechanical and electrical properties.

Figure 4d–f present the response times for the 6 phr of MoS_2_-based composites under 10–30%. These results also show the shortest response time at 20% strain. These results could be influenced by the viscoelasticity behavior, which can influence the mechanical response. Moreover, the quality of filler dispersion, which influences the mechanical and electrical response, is important [61]. These factors are influenced by the magnitude of the compressive strain, which correlates with the optimum packing fraction of filler particles at 20% strain. Such features indicate a short response time at 20% strain. It is also proposed that larger or more rapidly applied loads typically result in quicker, more pronounced responses. However, in our case, we test the response time under the same magnetic load under a 1 kN load cell and a similar strain rate. This assists us in understanding the influence of the filler type and strain on the response behavior. The response time is an important factor for various applications [16]. For example, the response time is critical for effective energy dissipation and noise reduction. Moreover, the response time is beneficial for sensor applications, where a rapid electrical response to mechanical stimuli is critical. Therefore, the response time is essential in developing efficient and reliable rubber-composite-based multifunctional products [62].

Experiments were performed to obtain the optimum packing fraction for the filler particles in the composites. The results showed that the optimum packing fraction was 30% for the compressive cyclic mechanical tests, 20% for the response time in the electromechanical tests, and 30% for the electromechanical stability tests. The role of the packing fraction is related to filler networking and percolation. At low filler content and strain, the packing fraction is low, and, at higher filler content, the filler starts to aggregate. This aggregation leads to a reduction in the mechanical and electromechanical properties. However, at certain filler content and strain, the properties are the best, which refers to the optimum packing fraction or filler percolation at this filler content and strain.

### 3.5. Mechanical and Electromechanical Stability Tests

Achieving mechanical and electromechanical stability is critical in terms of using the composites in sensors and energy harvesting applications. Such tests help to assess how these rubber composite materials respond to mechanical forces, electrical inputs, and environmental changes over time [63]. These tests ensure that these composite materials can withstand mechanical stresses and strains without failure. These tests are also useful for safety and reliability in various applications, like structural integrity for automotives and as a source of energy harvesting [64]. Moreover, stability tests ensure the consistent performance of these composites under mechanical stress and electrical inputs for crucial precision applications. Most importantly, stability tests ensure the reliability of energy production due to these composites’ high dielectric and piezoelectric properties [65].

**Figure 5:**
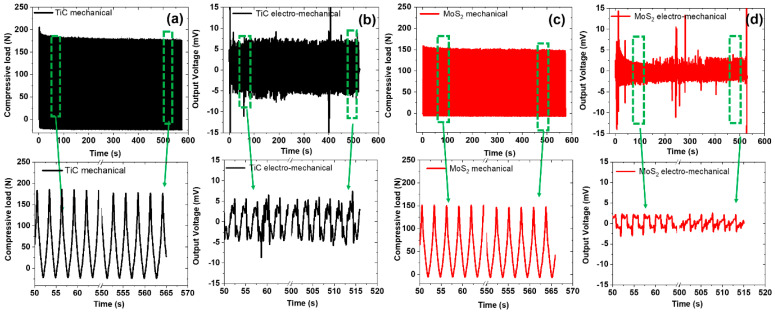
Mechanical and electromechanical stability at 30% compressive cyclic strain for (**a**,**b**) TiC-based composites; (**c**,**d**) MoS_2_-based composites.

With the above points in mind, mechanical and electromechanical stability tests were performed and are presented in Figure 5. Cylindrical samples were subjected to 30% compressive strain at 6 phr for the TiC- and MoS_2_-based composites. The results show that the mechanical and electromechanical stability is good, and this makes these composites useful for multifunctional applications. The key aspects of mechanical stability and electromechanical stability include fatigue resistance [66]. For example, it ensures the ability to endure repeated loading cycles without significant degradation. The results in Figure 5a–d show that the composites have robust degradation resistance. For example, their initial and final cycles show negligible degradation under continuous compressive cycling [67]. Moreover, the electromechanical properties include the interaction between mechanical deformation and electrical behavior (Figure 5b,d). Various factors influence the fatigue resistance of composites. These factors are the nature of the filler and rubber matrix used, the fabrication method, and the type of strain. Other notable factors are the piezoelectric effect, dielectric properties, and electrical conductivity [68]. Among them, the dielectric properties, such as the ability of a composite material to store and dissipate electric energy, are most critical. Similarly, the mechanical stability is mainly influenced by the filler dispersion and its orientation, the type of polymer matrix, the filler concentration, interfacial bonding, and the method of processing [69]. Almost all of these factors are critical in influencing the mechanical stability. Hence, the favorable mechanical stability depicted in Figure 5a,c shows that the composites prepared in this work have a balance of these factors. Overall, optimizing these properties can be achieved through appropriate material selection, design, and testing. Through these factors, engineers can create advanced composites tailored to meet specific requirements.

The main mechanism involved in ensuring mechanical and electromechanical stability is the high fracture toughness of the fillers used, as reported by Alam et al. [70]. Higher fracture toughness impacts the composites by providing high crack resistance, high durability, and safety. Most often, higher crack resistance leads to better mechanical stability. Moreover, this property is essential in maintaining the structural integrity under mechanical loads. In addition, improved fracture toughness allows for the better distribution of mechanical and electrical stresses within the material, reducing the likelihood of localized failure points and enhancing the overall stability. Thus, the composites studied in this work exhibit high mechanical and electromechanical stability, as shown in Figure 5.

### 3.6. SEM for Study of Filler Dispersion

It is well established that SEM is a powerful tool for the examination of the surface morphology and filler dispersion in composites. The fillers are added to the rubber matrix to improve its mechanical and electrical properties. Moreover, the dispersion of these fillers within the composite matrix significantly affects such properties [61]. Uniform filler dispersion leads to improved performance and consistency. However, the agglomeration or poor dispersion of filler particles can result in weak points and reduced effectiveness [71]. Moreover, SEM helps in assessing the uniformity of dispersion at the micro- and nanoscale, which is vital in achieving the desired electrical and mechanical properties.

**Figure 6:**
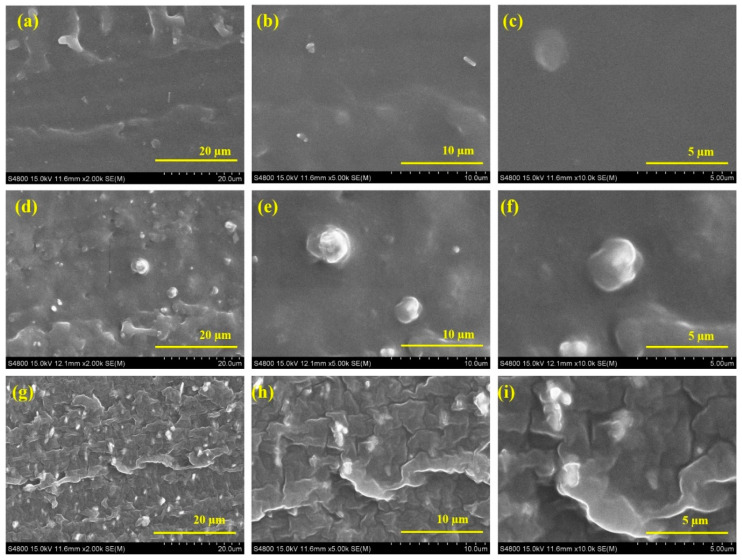
Filler dispersion of different composites at different magnifications: (**a**–**c**) control sample; (**d**–**f**) TiC-based composites, (**g**–**i**) MoS2-based composites.

With these points in mind, Figure 6a–c provide the SEM micrographs of the control sample without any filler. SEM is performed on these unfilled samples at both low and high resolutions. The study of the microstructure and surface characteristics of unfilled composites is crucial. It helps in understanding their mechanical properties, failure mechanisms, and performance under different conditions [72]. SEM is also used in studying the topological features of composites. SEM shows that the surface topology is smooth and influencing properties such as friction, wear resistance, and adhesion are absent. Moreover, the SEM of the unfilled composites shows the absence of surface defects, cracks, and voids, which might affect the composite material’s performance. Similarly, Figure 6d–f show the dispersion of TiC particles at 6 phr in the SR matrix. Generally, the filled composites are engineered to combine the beneficial properties of the matrix material with those of the fillers. The dispersion, distribution, and interaction of the fillers within the matrix critically influence the overall performance of the composite [73]. SEM analysis helps in understanding these factors, which are crucial in optimizing the composite material’s properties for specific applications. The low-resolution images show that the TiC particles are uniformly distributed within the rubber matrix. These features result in the improved mechanical and electromechanical properties seen in Figure 1, Figure 2, Figure 3, Figure 4 and Figure 5 in the above sections. Moreover, in the high-resolution images, the interfacial adhesion between TiC and the rubber matrix was analyzed. The SEM images show good interfacial adhesion and therefore good filler–rubber compatibility. Moreover, there was no evidence of surface defects, cracks, or voids in the filled composites. The SEM images also show that no fracture surfaces were found, providing insights into how TiC influences crack propagation and the failure mechanisms. These features indicate robust resistance against fatigue. These results further support the good mechanical and electromechanical stability of the composites under continuous compressive mechanical deformation, as shown in Figure 3 and Figure 5 above.

Similarly, Figure 6g–i show the SEM of the MoS_2_-filled composites at 6 phr loading. The results show that the MoS_2_ particles are uniformly distributed within the rubber matrix. They therefore support the good properties and stability against fatigue of the tested samples. The results also show how MoS_2_ influences the stress within the composite, contributing to the overall toughness or brittleness of the composite [9]. Moreover, the high-resolution image justifies the good interfacial interactions between the MoS_2_ particles in the rubber matrix. Moreover, there was no evidence of surface defects, cracks, or voids within the filled composites. Therefore, these results further validate the good filler–rubber compatibility that results in robust performance.

### 3.7. Prediction of Modulus through Theoretical Modeling

The validation of experimental data through theoretical models is well documented in research. The validation of the modulus of a filled rubber composite is crucial in designing materials with specific mechanical characteristics for diverse applications like energy harvesting. The prediction of the modulus through different models is useful for several reasons [74]. These are as follows: (a) it helps in designing composites with tailored mechanical properties for specific applications of interest, (b) it ensures that the composite will perform as expected under operational conditions, and finally (c) it ensures cost-effectiveness by reducing the need for extensive experimental testing by providing reliable theoretical predictions. Various theoretical models are used to predict the modulus. Among them, Guth-Gold–Smallwood equations and the Halpin–Tsai model are highly useful for filled rubber composites [75,76]. These models strictly depend on the aspect ratio of the filler, the modulus of the control sample, and finally the filler volume fraction in the composites. For example, the equation of the Guth-Gold–Smallwood model [75] is

E_TiC_ = E_o_ (1 + 0.67f_TiC_ϕ_TiC_) (1)

EMoS_2_ = E_o_ (1 + 0.67fMoS_2_ϕMoS_2_) (2)

Similarly, the Halpin–Tsai model [76] that is useful for prediction is

E_TiC_ = E_o_ [(1 + 2 f_TiC_ϕ_TiC_)/(1 − ϕ_TiC_) (3)

E MoS_2_ = Eo [(1 + 2 f MoS_2_ϕ MoS_2_)/(1 − ϕ MoS_2_) (4)

**Figure 7:**
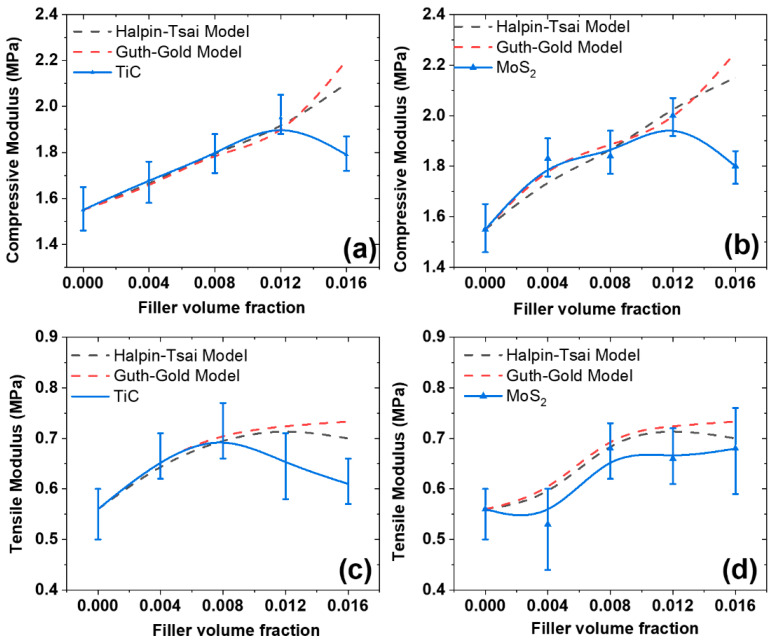
Theoretical models for prediction of composites: (**a**,**c**) TiC-based composites; (**b**–**d**) MoS_2_-based composites.

Here, “E” is the predicted modulus of the composite, “E_o_” is the modulus of the control sample, “f” is the aspect ratio of the filler, and “ϕ” is the volume fraction of the composite. The results presented in Figure 7a,b show that the models agree well with the literature up to 6 phr and then deviate from the experimental values. This deviation could be due to the partial aggregation of the filler particles in the experimental data. Due to aggregation, the properties fall after 6 phr loading for both the TiC- and MoS_2_-filled composites. Several factors influence accurate modulus prediction. These are filler dispersion, the filler morphology, the properties of the rubber matrix, the volume fraction of the fillers, and interfacial bonding [77]. Among them, the filler properties include the modulus, shape, size, and aspect ratio of the filler considered. Similarly, Figure 7c,d show that the model agrees well up to 6 phr for TiC and 8 phr for the MoS_2_-filled composites. The results indicate that the experimental data are influenced by uniform filler dispersion up to 6 phr for TiC and then slight aggregation occurs at 8 phr for the TiC-filled composite. Moreover, the MoS_2_-filled composites show fair agreement with the predicted models until 8 phr loading. However, the model assumes uniform filler dispersion and perfect interfacial bonding.

These features are difficult to achieve experimentally at higher loadings like 8 phr of TiC, which results in deviations in the experiments from the predicted models. Filler dispersion requires uniform filler distribution with minimum aggregates to achieve good predictions. Finally, the strong interfacial bonding between the matrix and fillers ensures efficient stress transfer, enhancing the composite’s modulus [78]. Overall, predicting the moduli of rubber composites through theoretical modeling is essential in designing materials with the desired mechanical properties. By understanding and applying various models, researchers and engineers can develop composites that meet specific performance requirements.

### 3.8. Calculation of Reinforcing Factor and Reinforcing Efficiency

The reinforcing factor and reinforcing efficiency measure the increase in the stiffness or modulus due to the addition of fillers in rubber composites. The reinforcing factor is typically expressed as the ratio of the composite’s modulus to that of the pure rubber matrix [79]. For example,

(5) $$R.F.=\frac{EF}{Eo}$$

**Figure 8:**
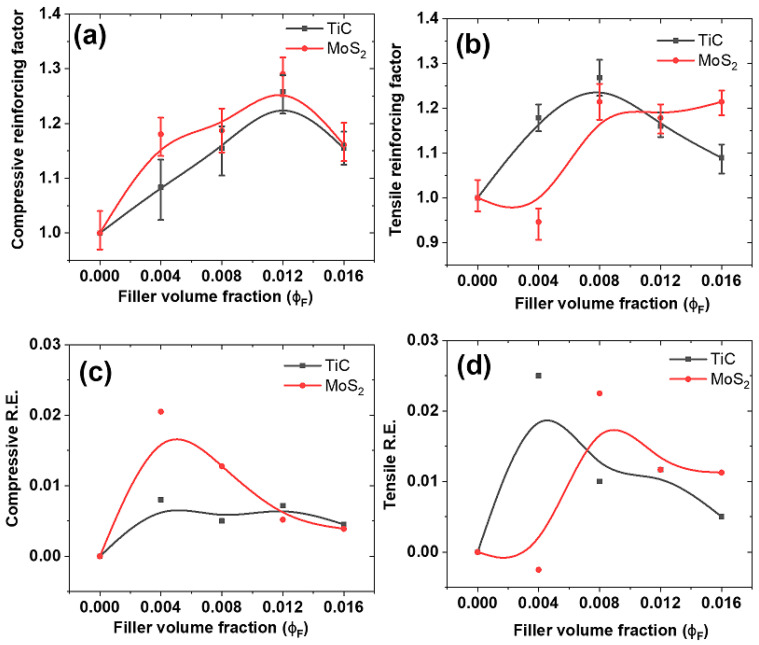
(**a**,**b**) Reinforcing factors of the composites; (**c**,**d**) reinforcing efficiency of the composites.

Here, R.F. is the reinforcing factor, E_F_ is the modulus of the filled composite, and E_o_ is the modulus of the unfilled rubber. Figure 8a,b show the behavior of the R.F. of the TiC and MoS_2_-based composites. The results indicate that the R.F. increases with increasing filler content until 6 phr and then falls. The increasing R.F. indicates that the addition of the filler provides reinforcement in the composite. The fall in the R.F. after 6 phr could be due to the partial aggregation of the filler particles in the composite. Various factors affect the R.F. in composites, such as the type of filler and rubber matrix and morphological features like the shape, size, and aspect ratio of the filler [28]. Filler dispersion is also proposed to influence the R.F. of composites. The main mechanism behind the reinforcing effect of fillers is filler–rubber bonding, which enhances the stress transfer from the rubber matrix to the fillers. The calculation of the R.F. is useful as it helps in designing composites with tailored mechanical properties for specific applications of interest. However, there are some challenges [80], such as (a) developing more accurate models to predict the reinforcing factors considering complex interactions between the fillers and the matrix; (b) exploring new types of fillers and their hybrid systems to obtain high reinforcement; (c) obtaining sustainable and eco-friendly fillers that reduce the environmental impact without compromising the performance; and (d) developing a new method of fabrication to obtain better filler dispersion and improved interfacial adhesion and compatibility between the filler particles and rubber matrix.

In the same way, the reinforcing efficiency was determined by the following equations [81]:

(6) $$R.E.at\;compressive\;strain=\frac{\sigma \left(30\%\right)filled-\sigma \left(30\%\right)unfilled}{wt\%\;of\;filler}$$

(7) $$R.E.at\;tensile\;strain=\frac{\sigma \left(100\%\right)filled-\sigma \left(100\%\right)unfilled}{wt\%\;of\;filler}$$

Here, R.E. is the reinforcing efficiency, σ (30%) is the stress of the filled composite at 30% compressive strain, σ (100%) is the stress of the filled composite at 100% tensile strain, and wt% is the weight percentage of the filled rubber. Hence, Figure 8c,d shows the R.E. of the filler particles in the composite. The results indicate that the R.E. depends upon the type of filler and the filler loading in the composites. For example, a higher R.E. indicates that the fillers are more effective in enhancing the modulus of the composite. Generally, a filler with a high reinforcing effect exhibits a higher R.E. Moreover, increasing filler content generally degrades the R.E. This is proposed to be due to the inverse proportionality of the filler content to the R.E. The R.E. quantifies the effectiveness of fillers in enhancing the mechanical properties of a composite [82]. As stated, it considers both the volume fraction of fillers and the resulting improvement in the modulus. Moreover, the R.E. provides insights into how well the fillers contribute to the composite’s stiffness relative to their concentration. Therefore, the R.E. is a vital metric in evaluating and optimizing the performance of rubber composites. By understanding and calculating these parameters, we can design composites with enhanced mechanical properties tailored to specific applications.

### 3.9. Real-Time Monitoring of Human Motion

The real-time monitoring of human motion involves monitoring mechanical deformation through employing smart technology. When coupled with energy harvesting technologies, this monitoring can capture and convert kinetic energy from human activity into electrical energy. This technology can be applied in various fields, such as sports training, physical therapy, or fitness practices [83]. The main aim of such technology is to enhance performance, prevent injuries before they occur, and promote correct movement patterns.

**Figure 9:**
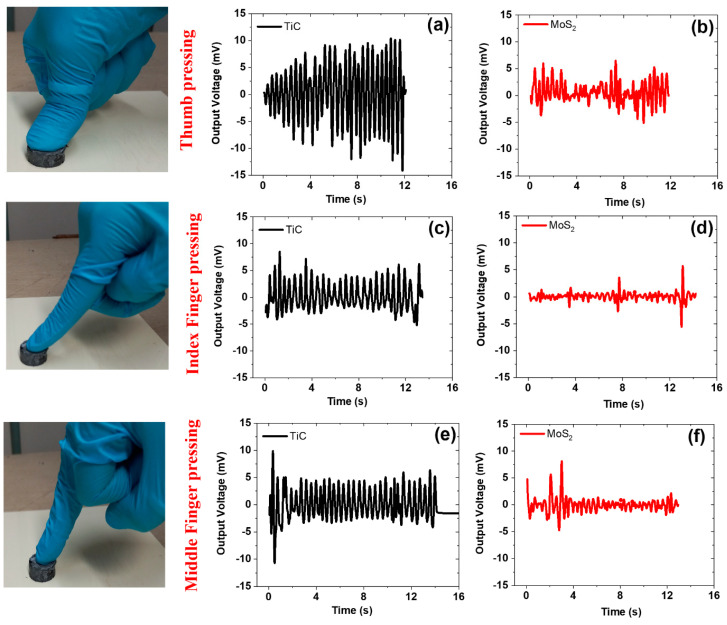
Real-time monitoring of the composites: (**a**,**b**) thumb pressing; (**c**,**d**) index finger pressing; (**e**,**f**) middle finger pressing.

The various applications for real-time monitoring technology include sports monitoring, health monitoring, fitness tracking, and physical therapy. With the above aspects in mind, Figure 9a–f present the behavior of energy generation through different types of human motion, like thumb pressing and finger pressing. The results show that thumb pressing is more efficient than finger pressing for all composites. This is due to the large contact area of the thumb, which provides a larger magnitude of strain than finger pressing [84].

Moreover, the TiC-based composites exhibit a higher output voltage than the MoS_2_-filled composites. These results agree with the electromechanical properties in Figure 3 and Figure 5 above. The main mechanism behind this technology involves piezoelectricity. For example, these composite materials generate an electrical charge in response to mechanical stress. Common applications include smart wristbands, thumb pressing, smart rings, and smart textiles. This technology has wide-ranging usefulness. For example, it reduces the dependence on batteries and external power sources, promoting sustainable energy solutions. Moreover, it enables continuous health monitoring, thereby providing valuable data for medical diagnosis and fitness tracking [85]. However, there are some challenges with this technology, such as poor efficiency, data accuracy, and durability. Overall, this technology represents a promising route for wearable technology and sustainable energy solutions. By capturing and converting kinetic energy from daily activities, these systems can power a wide range of devices, enhancing their functionality and sustainability [86].

## 4. Conclusions

Composites based on TiC, MoS_2_, and silicone rubber were successfully fabricated through solution mixing and their multifunctional aspects are detailed below.

(1) TiC and MoS_2_ act as reinforcing agents, thereby improving the mechanical properties of the rubber composites. For example, the compressive modulus was 1.55 MPa (control) and increased to 1.95 MPa at 6 phr of TiC and 2.04 MPa at 6 phr of MoS_2_. In the same way, the stretchability was 125% (control) and increased to 153% at 6 phr of TiC and 165% at 6 phr of MoS_2_.

(2) The electromechanical properties were also greatly improved when using these composites. For example, at 30% strain, the output voltage was 3.5 mV at 6 phr of TiC and 6.7 mV at 6 phr of MoS_2_.

(3) The models employed in the present work agree well until 6 phr and then deviate from the experimental values. This deviation could be due to the partial aggregation of the filler particles in the experimental data. The reinforcing factor and reinforcing efficiency show that TiC and MoS_2_ as fillers exhibit outstanding reinforcing effects on the silicone rubber matrix, thereby improving the mechanical properties of the composites.

(4) Real-time monitoring reveals the biomechanical properties. The tests show that the output voltage is the highest for thumb pressing. For example, thumb pressing results in an output voltage of ~10 mV at 6 phr of TiC and 5.6 mV at 6 phr of MoS_2_.

## Figures and Tables

**Table 1:** Table of formulations.

Sample	Amount of SR (phr)	Amount of TiC (phr)	Amount of MoS_2_ (phr)	Amount of Curing Agent (phr)
Control *	100	-	-	2
TiC-based composites	100	2, 4, 6, 8 **	-	2
MoS_2_-based composites	100	-	2, 4, 6, 8 **	2

* Here, the control sample refers to the unfilled sample, which did not contain any filler and only a vulcanized silicone rubber matrix was present. ** These values correspond to the composite samples containing 2 phr, 4 phr, 6 phr, and 8 phr of both TiC and MoS_2_ as fillers in silicone rubber.

**Table 2:** Summary of mechanical properties.

Sample Details	Compressive Modulus (MPa)	Tensile Modulus (MPa)	Tensile Strength (MPa)	Fracture Strain (%)
Control	1.55 ± 0.08	0.56 ± 0.03	0.54 ± 0.04	133 ± 7
TiC—2 phr	1.68 ± 0.09	0.66 ± 0.05	0.61 ± 0.05	125 ± 6
TiC—4 phr	1.79 ± 0.08	0.71 ± 0.07	0.78 ± 0.05	165 ± 11
TiC—6 phr	1.95 ± 0.07	0.65 ± 0.05	0.82 ± 0.07	153 ± 9
TiC—8 phr	1.79 ± 0.07	0.61 ± 0.05	0.61 ± 0.06	123 ± 6
MoS_2_—2 phr	1.83 ± 0.09	0.53 ± 0.04	0.52 ± 0.04	139 ± 8
MoS_2_—4 phr	1.85 ± 0.08	0.68 ± 0.05	0.74 ± 0.07	144 ± 8
MoS_2_—6 phr	2.02 ± 0.09	0.66 ± 0.04	0.85 ± 0.09	165 ± 12
MoS_2_—8 phr	1.8 ± 0.07	0.68 ± 0.04	0.69 ± 0.07	129 ± 7

## Data Availability

The original contributions presented in the study are included in the article, further inquiries can be directed to the corresponding author.
